# Assessment of spatio-temporal variations of selected water quality parameters of Lake Ziway, Ethiopia using multivariate techniques

**DOI:** 10.1186/s13065-022-00806-0

**Published:** 2022-03-14

**Authors:** Dessie Tibebe, Feleke Zewge, Brook Lemma, Yezbie Kassa

**Affiliations:** 1 Department of Chemistry, College of Natural and Computational Sciences, University of Gondar, P. O. box 196, Gondar, Ethiopia; 2 Department of Chemistry, College of Natural and Computational Sciences, Addis Ababa University, P.O. Box 1176, Addis Ababa, Ethiopia; 3 Department of Zoological Sciences, College of Natural and Computational Sciences, Addis Ababa University, P.O. Box 1176, Addis Ababa, Ethiopia; 4 Department of Biology, College of Natural and Computational Sciences, University of Gondar, P. O. box 196, Gondar, Ethiopia

**Keywords:** Cluster analysis, Comprehensive pollution index, Factor analysis, Principal component analysis

## Abstract

Excess agrochemicals input from agricultural activities and industrial effluent around Lake Ziway catchment can pose a serious threat on the lake ecosystem. Lake Ziway is a shallow freshwater lake found in the northern part of the Ethiopian Rift Valley. It is characterized as semi-arid to sub-humid type of climate. Expansions of the flower industry, widespread fisheries, intensive agricultural activities, fast population growth lead to deterioration of water quality and depletion of aquatic biota. The spatial and temporal variations of selected water quality parameters were evaluated using multivariate techniques. The data were collected from nine sampling stations during dry and wet seasonal basis for analysis of fifteen water quality parameters. The physicochemical parameters were measured in-situ with portable multimeter and nutrients were determined by following the standard procedures outlined in the American Public Health Association using UV/Visible spectrophotometer. Mean nutrient concentrations showed increasing trend in all seasons. These sites were also characterized by high electrical conductivity and total dissolved solid (TDS). All the nine sampling sites were categorized into three pollution levels according to their water quality features using cluster analysis (CA). Accordingly, sampling sites Fb and Ketar River (Kb) are highly and moderately polluted in both seasons, respectively. On the other hand, sampling sites at the center (C), Meki river mouth (Ma), Ketar river mouth (Ka), Meki River (Mb), Korekonch (K_o_) and Fa in dry season and Ka, C, Ma, Ko, Bulbula river mouth (B) and Fa during wet season were less polluted. Principal component analysis (PCA) analysis also showed the pollutant sources were mainly from Fb during dry season Mb and Kb during wet season. The values of comprehensive pollution index illustrated the lake is moderately and slightly polluted in dry and wet seasons, respectively. Comparatively, the pollution status of the lake is high around floriculture effluent discharge site and at the two feeding rivers (Kb and Mb) due to increasing trends in agrochemical loads. In order to stop further deterioration of the lake water quality and to eventually restore the beneficial uses of the lake, management of agrochemicals in the lake catchments should be given urgent priority.

## Introduction

Water pollution is one of the critical issues in environmental conservation. Freshwater resources have been of great importance to both natural ecosystems and human development. They are essential for agriculture, industry and human existence in general. The health of aquatic ecosystems is dependent on the presence of the right proportions of nutrients and requires succession of major nutrients in the water and sediment [1]. Appropriate assemblage of the nutrients ensures the status of water quality in any ecosystem and provides significant information about the available resources for supporting life [2].

Nitrogen and phosphorus (and silicon too for diatom species) are typical limiting nutrients influencing primary production. Nutrients occur in many different forms and only bio-available forms such as nitrate, nitrite, ammonia, orthophosphate and soluble reactive silica can be utilized directly by phytoplankton. Other forms of nutrients, however, can become bio-available through desorption, dissolution and biomass turnover. Nutrients in the water body may originate from weathering of bedrock, atmospheric precipitation, terrestrial input, storm water runoff, sewage effluent and agricultural discharge [3, 4].

Nutrient enrichment of lakes is considered to be one of the major environmental problems in many countries especially in developing ones [5]. In recent decades, population growth, agricultural practices and sewage runoff from urban areas have increased nutrient inputs many folds to the level of their natural occurrence, resulting in accelerated eutrophication [5, 6]. Many urban and rural lakes have vanished under this pressure with worldwide environmental concerns [7]. The evaluation of water quality in freshwater lakes is indispensable due to its immense significance in terms of ecological services and livelihood perspectives. Anthropic pressures, however, in the form of rapid urbanization, excessive use of pesticides and fertilizers, land use and climate change are diminishing the water quality, which necessitates better insights into pollution variability and its controlling measures [8, 9].

Multivariate statistical techniques have been widely adopted to analyze and evaluate surface and freshwater water quality, and are useful to verify temporal and spatial variations caused by natural and anthropogenic factors linked to seasonality [7, 10]. Although the numerous management challenges, the multivariate techniques have a limited usage in the assessment of water quality in many lakes in developing countries including Lake Ziway.

Despite its ecological and economic importance, both locally and globally, Lake Ziway has been facing alarming environmental degradation and loss of biodiversity due to the pressure of human land-use and climate change. Substantial increases in water pollution, largely from the discharge of untreated municipal and industrial waste and high sediment load from agricultural fields caused by unchecked erosion in upper catchments, are the major causes. The main significances of the study will address for discouraging farming activities along the lakeshore; to set a standardized buffer zone around the lake shore; all the free access policy (no ownership scenario) to water bodies will have had to change for Lake Ziway by giving concession rights to users with the appropriate environmental regulatory protocols and it also helps to develop mitigation and restoration strategies for the lake and aquatic ecosystems in Ethiopia.

Moreover, pollution of freshwater with potential contaminants due to natural phenomena and anthropogenic activities are of great concern worldwide [9]. The freshwater lakes are susceptible to chemical contaminations as they are stagnant in nature [9]. Systematic studies on geochemical variability’s and inference of natural and anthropogenic factors are crucial to explain and protect the water quality in the lake ecosystem. However, studies on the comprehensive spatio-temporal variations and the systematic identification of the potential pollution sources of Lake Ziway water qualities were very limited. Thus, reliable information on water quality and pollution sources is important for effective lake water management. Therefore, the objective of this study is to assess the spatio-temporal variations of selected water quality parameters of Lake Ziway using Multivariate Techniques.

## Materials and methods

### Description of the study area

**Figure1:**
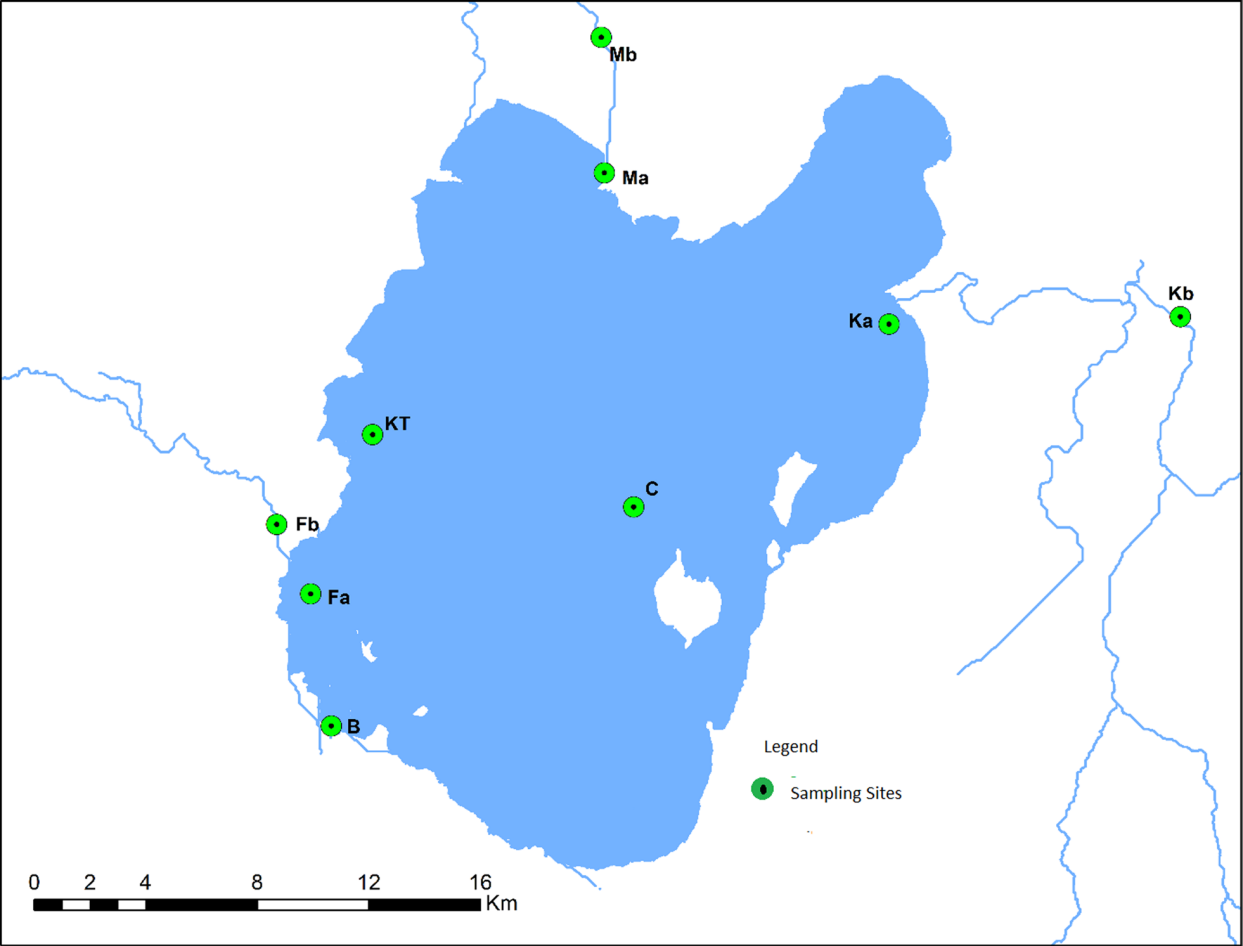
Location and bathymetric Map of Lake Ziway and its tributaries with the sampling sites

Lake Ziway is shallow freshwater located in the most northern section of the Ethiopian Rift Valley. The region is characterized as semi-arid to sub-humid type of climate and has mean annual precipitation varying between 650 and 1200 mm and mean annual temperature between 15 and 25 °C [11]. During the last few decades, Lake Ziway has begun to show reduction in its water level because of some climatic factors and excessive water abstraction for irrigation, municipals and industrial purposes [12]. The lake is fed primarily by Meki and Ketar Rivers and drained by the Bulbula River. The lake's catchment has an area of 7025 km^2^ with the town of Ziway lying on the lake's western shore [12]. Lake Ziway is situated at 1636 m above sea level and at 08º01’N and 38º47’E (Fig. 1 and Table 1) in a complex geological arrangement of sedimentary deposits.

**Table 1:** Geographic coordinates of the sample points

Sampling site description	Abr	North	East	Elevation (m)
Floriculture effluent	Fb	07º54.715'	038º44.020'	1642
Floriculture after mixing	Fa	07º54.79’	038º144.111’	1639
Bulbula River mouth	B	07º53.943'	038º44.134'	1641
Ketar River mouth	Ka	07º55.398'	038º52.086'	1640
Ketar River at Abura Town	Kb	08º02.019'	038º49.340'	1646
Meki River at Meki Town	Mb	08º03.019'	039º01.144'	1673
Meki River mouth	Ma	08º03.379'	038º56.459'	1633
Korekonch	Kt	07º55.494'	038º43.697'	1637
Central station	C	07º55. 49’	038º52.934	1635

The lake provides water for domestic use and shares the same water table with key groundwater aquifers that provide borehole water supply for the rapidly-expanding human population in Ziway town and surrounding areas. Currently, the population of Lake Ziway catchment is about 2 million and about 1.9 million livestock [13, 14]. The fishery of the lake is also an important source of livelihood to scores of fishermen and their families and provides the sources of food to many families within the lake basin and beyond. Tourism is also a major activity in the area due to the presence of hipotanuse, scenery Islands with monasteries, bird sanctuaries and the presence of rich tropical related biodiversity [15]. Other socio-economic activities conducted along the lake’s shore include livestock production and small-scale farming [16].

Agriculture is the most dominant land use system contributing to the livelihoods of the majority of the catchment population. The agricultural sector is characterized by small-scale subsistence-based farming and rising of livestock. About 74.3% of the total land-use types within the catchment are agricultural lands. Lake Ziway water demands have massively increased, along with increased population and intensification of agriculture since the end of the last decade [17].

### Chemicals, reagents and standards

Analytical reagent grade sodium hydroxide, concentrated hydrochloric acid, concentrated sulfuric acid, concentrated phosphoric acid, anhydrous sodium sulfate, ammonium persulfate, potassium persulfate, Phenol, sodium nitroprusside, sulfanilamide, *N*-(1-naphthyl)-ethylenediamine dihydrochloride, Potassium chloride, sodium salicylate, potassium sodium tartarate, boric acid, potassium antimony tartrate, ammonium molybdate, ascorbic acid ethanol (99.99%), phenolphthalein, methyl orange. All chemical and reagents are products of Sigma-Aldrich, Germany.

### Apparatus and equipment

UV–Visible Spectrophotometer (Jenway 6405, UK); Kjeldahl apparatus (Gallenhamp, USA); Oven dry (Binder, Germany); Turbidimeter (T-100, Singapore); portable multi meter (HACH MM150, China) were used in the experiments.

### In-situ measurements

All field equipments were calibrated according to the manufacturer’s specifications**.** Temperature, pH, electrical conductivity, total dissolved solids, and dissolved oxygen (DO) were measured with a portable ion meter (HACH™ model150 made in Spain. Secchi depth (SD) was measured with a standard Secchi disk of 20 cm diameter.

### Sampling and laboratory analysis

The collection of data for analyses of major nutrients and physico-chemical water quality parameters in water samples in different depths at the selected sampling sites taken at selected seasons for two years. Nine representative sampling sites were selected purposefully based on access, safety, waste disposal activities, lake inflow and outflow and geographical proximity. These sites were evenly distributed along the course of Lake Ziway.

Water samples were collected with a Van Dorn water sampler from different depths of the entire water column at 1 m intervals and mixed in equal proportions to produce composite samples. The collected water samples were kept in precleaned polyethylene plastic bottles for nutrient analysis following the standard guideline values [18]. All water samples were stored in insulated dark ice boxes and taken on the same day to the laboratory.

For quality control and quality assurance, the standard operating procedures were strictly followed during sampling and laboratory analysis as directed by [18]. In order to avoid contamination, powder-free nitrile exam gloves and mask was used during the sample collection and testing. At each sampling site, three water samples, i.e., at left bank, middle, and right bank of the lake, were taken and mixed before a composite sample was prepared. The sample bottles were prerinsed three times with the same water before the final sample was acquired. Before the in situ measurements, the instruments were properly calibrated. Triplicate samples were run, and the average recovery of quality control analysis was 99 ± 4%, indicating the good quality of the data. In addition, four blank samples of deionized water filtered through 0.45-μm poly tetrafluoroethylene (PTFE) disk syringe filter, filled in high-density polyethylene (HDPE) bottles, sealed with Parafilm were collected in the field and kept in the same environment with other water samples. The results of these field blank samples showed negligible contamination during the sampling, filtering, and storage processes, as the values of most hydrochemical variables were below the detection limit [19–21].. The average analytical precision for nutrients was better than 2%. The alkalinity as HCO_3_^−^ was estimated by charge balance [19]. To interpret the data and develop a conclusive understanding of the geochemistry of Lake Ziway water quality, a series of statistical tests were performed using an IBM SPSS 22.0 [20]. These tests include a normality test, descriptive statistics (mean, max, min, SD etc.), Spearman correlation, and principal component analysis and factor analysis (PCA/FA) [19, 20]. The normality test and correlation analysis were performed by considering all of the parameters to predict the degree of dependent of one variable on others with a correlation significance level of 0.01. PCA/FA was applied to group the changing patterns of physico-chemicals parameters and nutrients in order to explain the fluctuation in dataset with minimum loss of original information. PCA/FA is attained by analyzing the correlation matrix and transforming the original variables to uncorrelated ones, commonly called varifactors (VFs) [19]. Additionally, the eigen values in PCA/ FA define how much variance is present in associated VFs. The VF that holds the maximum eigenvalue is found to have the most co-variability [22, 23]. Suitability of the dataset for PCA/FA was tested by using the Kaiser–Meyer–Olkin (KMO) and Bartlett’s sphericity methods which is run prior to PCA/FA.

### Chemical analysis

Concentrations of inorganic nutrients (NO_2_-N, NO_3_-N, PO_4_-P, NH_3_-N, total phosphorus (TP), total nitrogen (TN), total inorganic nitrogen (TIN) and soluble reactive silica (SiO_2_-Si) were determined for all samples following the standard procedures outlined in [18]. Table 2 summarizes the analytical methods for surface water samples.

**Table 2:** **S**ummary of analytical methods used for surface water sample (APHA [18])

Parameter	Method	Description
Total alkalinity	APHA 2320 B	Titrimetric
pH	Membrane Electrode	Portable HACH™ model 150
EC	Membrane Electrode	Portable HACH™ model 150
TDS	Membrane Electrode	Portable HACH™ model 150
Temperature	Membrane Electrode	Portable HACH™ model 150
Ammonia	APHA4500-NH_3_ C	Spectrophotometric, Phenate
Nitrate	Yang et al*.*, 1998	Spectrophotometric, sodium salicylate
Nitrite	APHA4500- NO_2_^−^ A	Spectrophotometric, Colorimetric
TN	APHA4500- N C	Spectrophotometric, Kjeldahl method
Phosphate	APHA4500-P C	Spectrophotometric, Ascorbic Acid
TP	APHA4500-P C	Spectrophotometric, Persulfate digestion method, then Ascorbic acid method
Secchi depth	Lind	Field equipment
Dissolved oxygen	Membrane Electrode	probe method (YSI model 58)
Silica	APHA4500-SiO_2_	Spectrophotometric, Molybdosilicate Method

### Multivariate statistical methods

Lake water quality data sets were subjected to three multivariate techniques: cluster analysis (CA), principal component analysis (PCA) and factor analysis (FA) [24]. All statistical analyses were performed using the SPSS statistical software (Version 20) and PAST statistical software (Version 1.93) [24].

### Cluster analysis

CA classifies objects, so that each object is similar to the others in the cluster with respect to a predetermined selection criterion. Hierarchical agglomerative clustering is the most common approach, which provides intuitive similarity relationships between any one sample and the entire data set and is typically illustrated by a dendrogram (tree diagram). The dendrogram provides a visual summary of the clustering processes, presenting a picture of the groups and their proximity with a dramatic reduction in dimensionality of the original data [7, 25, 26]. In this study, hierarchical agglomerative CA was carried out on the normalized data by means of Ward’s method, using squared Euclidean distances as a measure of similarity.

### Principal component analysis (PCA)/factor analysis (FA)

In this research, PCA was applied to summarize the statistical correlation among water quality parameters. The concentrations of physico-chemical parameters and nutrients tend to differ greatly; as such, the statistical results should be highly biased by any parameter having a high concentration. Thus, each water quality parameter was standardized before PCA the analysis was performed in order to minimize the influence of different variables and their respective units of measurements. The calculations were performed based on the correlation matrix of chemical components, and the PCA scores were obtained from the standardized analytical data [25, 27].

### Comprehensive evaluation of water quality in the lake

A comprehensive pollution index method has been applied to evaluate water quality qualitatively in many existing studies. The comprehensive pollution index can be calculated as follows [28]:

$$\mathrm{P}=1/n \sum\limits_{n=1}^{n}\left(\mathrm{Ci}/\mathrm{Si}\right)$$

where P is comprehensive pollution index, C_i_ is the measured concentration of the pollutant (mg L^−1^), S_i_ represents the limits allowed by the State Environmental Protection Administration (SEPA) in the particular country for water quality standard, and n is the number of selected pollutants [24, 28, 29]. Ultimately, the values determined for P could be used to classify the water quality level of the lake (Table 3).

**Table 3:** Standard of surface water quality classification (WHO, 1996)

Comprehensive pollution index (P)	Water quality level
≤< 0.20	I cleanness
0.20 to 0.40	II sub-cleanness
0.41to1.00	III slight pollution
1.01 to 2.00	IV moderate pollution
≥ 2.01	V sever pollution

### Statistical analysis

Different procedures of statistical analyses were used to analyze the data. Analysis of variance (ANOVA) was conducted to test the differences between, and within, sampling sites at 95% confidence interval using SPSS (version 20) software (Chicago, USA). The differences between sites were examined to determine the spatial variation while the differences within seasons addressed the temporal variation for water samples. All correlations were considered statistically significant when the significance level was p < 0.05.

## Results

### Spatial and temporal variations in Physico-chemical water quality parameters

The average spatio-temporal values of physico-chemical water quality parameters in dry and wet seasons are given separately in Tables 4 and 5, respectively. The surface-water temperature measured in the study site ranged from 19.0 to 27.0 °C and 18.0 to 27.0 °C in dry and wet seasons, respectively. The highest values were measured at B and Ka and the lowest values were measured at C and Fb during dry and wet seasons, respectively.

**Table 4:** Mean, mean standard error and range of the physicochemical parameters in dry season (Temp for temperature in ^o^C; DO for dissolved oxygen in mg L^−1^; pH for H^+^ concentration; EC for electrical conductivity in μS cm^−1^; TDS for total dissolved solids in mg L^−1^; SD for secchi depth in cm; TA for total alkalinity, mg L^−1^; Turbidity in NTU)

Site		Temp	DO	pH	EC	TDS	SD	TA
B	x̄ ± Std. Err	24.8 ± 1.3	7.3 ± 1.8	8.3 ± 0. 3	385 ± 38	248 ± 24	24.6 ± 0.5	314 ± 41
	Range	21–28	4.8–12.4	8–9	289–521	189–335	23–26	216–425
C	x̄ ± Std. Err	21.2 ± 1.3	5.2 ± 1.1	8.1 ± 0.2	408 ± 43	268 ± 33.7	27.7 ± 1.3	225 ± 22
	Range	18–25	4–8.4	8–9	337–558	215–393	23–30	184–300
Fa	x̄ ± Std. Err	23.8 ± 1.0	6.8 ± 1.7	8.13 ± 0.2	639.73 ± 114	423.8 ± 83	26 ± 0.7	320 ± 50
	Range	21–26	4.2–11.2	8–9	376–1028	249–720	24–28	200–425
Fb	x̄ ± Std. Err	23. 5 ± 1.4	6.2 ± 1.4	7.56 ± 0.1	1233.6 ± 107	789.6 ± 68.3	25.8 ± 0.6	463 ± 71.7
	Range	19–27	2.6–9.3	7–8	1050–1650	672–1056	24–27	200–625
Ka	x̄ ± Std. Err	21.3 ± 0.5	4.4 ± 1.5	8.1 ± 0.16	382.6 ± 39.4	237.8 ± 18.6	24.4 ± 1.8	24 ± 29
	Range	20–23	2.5–9.2	7–8	307–543	196–307	19–30	156–325
Kb	x̄ ± Std. Err	20.15 ± 0.2	5.5 ± 0.6	7.4 ± 0.1	170.7 ± 14.3	107.7 ± 9	21 ± .7	187 ± 44
	Range	20–21	4.0–7.0	7–8	134–204	86–130	19–23	104–275
Ko	x̄ ± Std. Err	24.2 ± 1.3	5.4 ± 1.3	7.4 ± 0.8	399 ± 14.4	254 ± 10.5	25.6 ± 1.1	330 ± 38
	Range	21–28	2.4–9.0	7–9	362–435	219–278	22–28	200–425
Ma	x̄ ± Std. Err	22.2 ± 0.7	4.4 ± 0.6	7.98 ± 0.2	404.1 ± 43	291.9 ± 37.7	27.8 ± 1.8	227.6 ± 26
	Range	21–25	3.3–6.5	8–9	333–576	213–403	22–31	160–300
Mb	x̄ ± Std. Err	22.9 ± 0.8	7.6 ± 1.6	7.95 ± 0.2	424 ± 625	267 ± 38	21 ± 0.71	263 ± 48
	Range	20–24	3.8–10	7–9	203–584	130–365	19.00	100–375

**Table 5:** Mean, mean standard error and range of the physicochemical parameters in wet season season (Temp for temperature in ^o^C; DO for dissolved oxygen in mg L^−1^; pH for H^+^ concentration; EC for electrical conductivity in μS cm^−1^; TDS for total dissolved solids in mg L^−1^; SD for secchi depth in cm; TA for total alkalinity, mg L^−1^; Turbidity in NTU)

Site		Temp	DO	pH	EC	TDS	SD	TA
B	x̄ ± Std. Err	22 ± 0.5	4.72 ± 0.4	8.59 ± 0.1	274.5 ± 9	175.7 ± 58.2	15.3 ± 0.3	207 ± 6.3
	Range	21.5–23	4.27–5.4	8.4—8.7	176—456	113–292	15–16	200–220
C	x̄ ± Std. Err	21.2 ± 0.3	4.75 ± 28	8.57 ± 0.1	218 ± 44	147.8 ± 37	17.3 ± 0.7	168 ± 6.1
	Range	20.7–22	4.4–5.3	8.4–8.7	173–306	110–222	16–18	160–180
Fa	x̄ ± Std. Err	22 ± 0.6	4.4 ± 0.3	8.78 ± 0.5	353 ± 89	226 ± 56	18.6 ± 0.7	172 ± 14
	Range	20.8–23	3.95–4.8	7.9–9.7	187–488	120–312	18–20	148- 196
Fb	x̄ ± Std. Err	21 ± 1.6	6.2 ± 0.7	8.53 ± 0.1	370 ± 98	216 ± 84	19.6 ± 0.7	250 ± 111
	Range	18–24	4.9–7.2	8.3–8.7	175–478	49–306	19–21	120–472
Ka	x̄ ± Std. Err	22 ± 0.3	3.1 ± 0.3	8.4 ± 0.1	230 ± 41.4	180 ± 32	16–1.2	117–35
	Range	20—27	2.4 -3.5	8.3–8.5	183–312	117–221	14–18	80–188
Kb	x̄ ± Std. Err	20 ± 0.2	2.6 ± 0.6	7.78 ± 0.2	101 ± 17.9	65 ± 11.7	17.3 ± 0.9	72.3 ± .3
	Range	20–21	1.4–3.6	7.5–8.3	65–120	42–78.3	16–19	72–73
Ko	x̄ ± Std. Err	22.5 ± 0.9	4.8 ± 0.3	8.7 ± 0.1	381 ± 101	234 ± 61	18.3 ± 0.9	175 ± 14.6
	Range	21–24	4.35–5.3	8.5–8.8	179–496	116–318	17.-20	146–190
Ma	x̄ ± Std. Err	23 ± 0.1	3.9 ± 0.1	8.6 ± 0.1	273 ± 49	153 ± 21.3	17 ± 1.2	130 ± 24.9
	Range	22–23	3.7–4.1	8.6–8.61	176–337	113–185	15–19	100–180
Mb	x̄ ± Std. Err	20.4 ± 0.7	4.8 ± 0.8	7.4 ± 0.5	120 ± 3	77 ± 1.9	18 ± 1.7	100 ± 20
	Range	19.5–22	3.4–5.8	6.5–8.3	115–126	73.7–80.4	15–21.0	60–120

The level of dissolved oxygen (DO) ranged from 2.42 to 12.4 mg L^−1^ and 1.4 to 7.2 mg L^−1^ in dry and wet seasons, respectively (Tables 4 and 5). The lowest values in both seasons were at K_o_ (2.4 mg L^−1^) in dry and K_b_ (1.4 mg L^−1^) in wet seasons where as the highest values were at B (12.4 mg L^−1^) in dry and F_b_ (7.2 mg L^−1^) in wet seasons, respectively. Whilst the pH values ranged from 7.0 to 9.0 and 6.5 to 9.7 in dry and wet seasons respectively (Tables 4 and 5).

The mean electrical conductivity (EC) values in the study sites ranged from 134.0 to 1650 μS cm^−1^ in the dry season and 65.0 to 488 μS cm^−1^ in the wet season. Kb showed the lowest mean EC (134 μScm^−1^) in dry and 65 μS cm^−1^ in wet seasons while site Fb recorded the highest mean values of 1650 μS cm^−1^ in dry and Fa mean values 488 μS cm^−1^ in wet seasons, respectively (Tables 4 and 5). Total dissolved solid (TDS) ranged from 119.77 to 746.80 mg L^−l^ with the lowest value was recorded in sampling site Kb and the highest value was recorded in sampling site Fb while in the wet season, it ranged from 129.5 to 547.76 mg L^−1^ at sites Mb and Fb, respectively (Tables 4 and 5). The mean SD values ranged from 0.20 to 0.22 m, with mean values of 0.21 m.

Total alkalinity (TA) in the study sites were ranged from 100 to 625 and 60 to 472 mg CaCO_3_ L^−1^ in dry and wet seasons, respectively (Tables 4 and 5). Mb showed the lowest mean TA 100 mg CaCO_3_ L^−1^ in dry and 60 mg CaCO_3_ L^−1^ in wet seasons while the highest mean values of TA was 625 and 472 mg CaCO_3_ L^−1^ in dry and wet seasons, respectively in sampling site Fb.

### Nutrients analyses

The spatial and temporal variations of nutrients are summarized in Tables 6 and 7. The mean NO_3_-N concentration ranged from 0.1 to 5.26 mg L^−1^ and 0.01 to 0.86 mg L^−1^ in dry and wet seasons, respectively. The highest mean NO_3_-N was recorded at Fb in dry and Kb in wet season while the lowest values were in Ma in both dry and wet seasons. NO_2_-N ranged from 0.06 to 2.89 mg L^−1^ in dry season and 0.20 to 1.8 mg L^−1^ in wet season during the study period (Tables 6 and 7). Low concentrations of NO_2_-N were at M_a_ in both dry and wet seasons whereas high concentrations were at F_b_ and K_b_ in dry and wet seasons, respectively (Tables 6 and 7).

**Table 6:** Mean, mean standard error and range of nutrient concentrations (mg L^−1^) measured in sampling sites at Lake Ziway in dry season (TP for total phosphorus in mg L^−1^; SRP for soluble reactive phosphorus in mg L^−1^; NO_2_-N for nitrite-nitrogen in mg L^−1^; NO_3_-N for nitrate-nitrogen in mg L^−1^; NH4-N for ammonia–nitrogen in mg L^−1^; TIN for total inorganic nitrogen in mg L^−1^; TN for total nitrogen in mg L^−1^; SiO_2_-Si for soluble silica in mg L^−1^)

Site		TP	PO_4_-P	NO_2_-N	NO_3_-N	NH_3_-N	TIN	TN	SiO_2_-Si
B	x̄ ± Std. Err	0.12 ± 0.02	0.06 ± 0.01	0.48 ± 0.20	0.17 ± 0.04	0.21 ± 0.05	0.85 ± 0.25	5.7 ± .25	46.2 ± 6.2
	Range	0.06–0.15	0.04–0.08	0.188–1.3	0.06–0.25	0.1–0.35	0.34–1.8	4.9–6.4	32–68
C	x̄ ± Std. Err	0.14 ± 0.02	0.05 ± 0.01	0.29 ± 0.05	0.26 ± 0.17	0.17 ± 0.03	0.72 ± 0.22	9.1 ± 0.65	46.8 ± 2.4
	Range	0.1–0.185	0.03–0.07	0.18–0.41	0.01–0.91	0.09–0.26	0.29–1.5	7.34–11.20	39.5–54
Fa	x̄ ± Std. Err	0.14 ± 0.02	0.05 ± 0.01	0.96 ± 0.22	0.38 ± 0.13	0.24 ± 0.04	1.6 ± 0.36	6.1 ± .6	46.8 ± 4.9
	Range	0.105–0.23	0.03–0.1	0.6–1.8	0.1–0.75	0.15–0.35	0.9–2.9	4.5–8.3	35–60
Fb	x̄ ± Std. Err	0.19 ± 0.05	0.08 ± 0.03	1.7 ± 0.44	0.58 ± 0.19	0.29 ± 0.05	2.6 ± 0.6	8.1 ± .56	91.39.8
	Range	0.05–0.32	0.04–0.16	0.72–2.89	0.08–0.97	0.15–0.42	1.0–4.2	6.5–9.8	56.9–114
Ka	x̄ ± Std. Err	0.13 ± 0.02	0.05 ± 0.01	0.35 ± 0.08	0.12 ± 0.02	0.22 ± 0.05	0.68 ± 0.10	7.1 ± 1.1	50.5 ± 6.7
	Minimum	0.09–0.19	0.04–0.07	0.155–0.64	0.08–0.18	0.10–0.4	0.40–0.95	5.0–11	40.1–77
Kb	x̄ ± Std. Err	0.17 ± 0.03	0.05 ± 0.01	0.34 ± 0.03	0.22 ± 0.05	0.34 ± 0.03	0.89 ± 0.08	9.2 ± 1.3	68 ± 7.8
	Range	0.08–0.24	0.04–0.09	0.24–0.42	0.07–0.35	0.24–0.42	0.76–1.2	14-Jul	43–88.7
Ko	x̄ ± Std. Err	0.12 ± 0.02	0.05 ± 0.01	0.32 ± 0.09	0.10 ± 0.03	0.2 ± 0.04	0.62 ± 0.14	9.7 ± .42	39 ± 3.4
	Range	0.07–0.19	0.04–0.07	0.05 ± 0.188	0.01–0.18	0.09–0.3	0.3–1.2	8.5–11	30–47
Ma	x̄ ± Std. Err	0.14 ± 0.02	0.05 ± 0.01	0.23 ± 0.08	0.10 ± 0.02	0.17 ± 0.03	0.55 ± 0.08	6.3 ± .49	49 ± 3.8
	Range	0.09–0.2	0.04–0.06	0.1–0.53	0.04–0.14	0.114–0.3	0.35–0.8	4.8–7.6	38–62
Mb	x̄ ± Std. Err	0.97 ± 0.75	0.06 ± 0.01	0.41 ± 0.17	0.28 ± 0.19	0.41 ± 0.17	1.3 ± 0.53	9.4 ± 1.2	61.3 ± 3.9
	Range	0.2–3.95	0.04–0.08	0.06–1.1	0.03–1.1	0.1–1.1	0.21–3.2	5.6–13	50.8–73

**Table 7:** Mean, mean standard error and range of nutrient concentrations (mg L^−1^) measured in sampling sites at Lake Ziway in wet season(TP for total phosphorus in mg L^−1^; SRP for soluble reactive phosphorus in mg L^−1^; NO_2_-N for nitrite-nitrogen in mg L^−1^; NO_3_-N for nitrate-nitrogen in mg L^−1^; NH4-N for ammonia–nitrogen in mg L^−1^; TIN for total inorganic nitrogen in mg L^−1^; TN for total nitrogen in mg L^−1^; SiO_2_-Si for soluble silica in mg L^−1^)

Site		TP(mg/L)	PO_4_-P	NO_2_-N	NO_3_-N	NH_3_-N	TIN	TN	SiO_2_-Si
B	x̄ ± Std. Err	0.35 ± 0.1	0.046 ± 0.01	0.47 ± 0.06	0.15 ± 0.03	0.11 ± 0.02	0.73 ± 0.08	5.13 ± 1.20	35.5 ± 11.60
	Range	0.21–0.42	0.04–0.06	0.35–0.53	0.08–0.20	0.07–0.13	0.57–0.86	2.8–7.00	12.37–47.30
C	x̄ ± Std. Err	0.38 ± 0.023	0.05 ± 0.01	0.33 ± 0.12	0.17 ± 0.12	0.09 ± 0.01	0.59 ± 0.24	6.02 ± 0.26	43.6 ± .72
	Range	0.34–0.41	0.03–0.08	0.21–0.57	0.05–0.41	0.08–0.10	0.352–1.1	5.60–6.5	42.9–45.1
Fa	x̄ ± Std. Err	0.29 ± 0.12	0.05 ± 0.01	0.74 ± 0.22	0.26 ± 0.12	0.09 ± 0.03	1.1 ± 0.33	7.0 ± 0.81	36 ± 7.5
	Range	0.18–0.52	0.04–0.07	0.43–1.2	0.03–0.39	0.03–0.14	0.48–1.62	5.6–8.4	22.2–47.7
Fb	x̄ ± Std. Err	0.42 ± 0.175	0.11 ± 0.04	0.89 ± 0.44	0.44 ± 0.19	0.09 ± 0.04	1.42 ± 0.62	6.66 ± 0.53	39.2 ± 26
	Range	0.17–0.75	0.04–0.16	0.34–1.8	0.17–0.80	0.02–0.15	0.75–2.7	5.6–7.4	5.8–90.5
Ka	x̄ ± Std. Err	0.23 ± 0.02	0.05 ± 0.01	0.84 ± 0.16	0.47 ± 0.04	0.09 ± 0.03	1.39 ± .18	7.6 ± 0.43	37.9 ± 4.68
	Range	0.20–0.27	0.04–0.07	0.5—1.1	0.4–0.54	0.03–0.12	1.1 ± 1.7	7–8.4	30.3–46.45
Kb	x̄ ± Std. Err	0.73 ± 0.27	0.06 ± 0.01	1.2 ± 0.20	0.86 ± 0.22	0.15 ± 0.10	2.2 ± .50	8.1 ± 0.86	38.15 ± 17.10
	Range	0.24–1.2	0.05–0.08	0.89–1.6	0.52–1.3	0.05–0.3	1.5–3.1	7–9.8	11.24–70
Ko	x̄ ± Std. Err	0.27 ± 0.08	0.05 ± 0.01	0.42 ± 0.12	0.20 ± 0.04	0.08 ± 0.02	0.7 ± 0.12	12 ± 1.9	34.3 ± 11
	Range	0.12–0.376	0.04–0.07	0.2–0.6	0.1–0.3	0.05–0.1	0.5–0.9	8.4–14	12.6–48.2
Ma	x̄ ± Std. Err	0.24 ± 0.04	0.06 ± 0.02	0.76 ± 0.06	0.23 ± 0.11	0.08 ± 0.02	1.06 ± 0.14	6.1 ± .55	42.62 ± 1.9
	Range	0.19–0.33	0.04–0.082	0.66–0.86	0.01–0.369	0.03–0.11	0.8–1.3	4.97–7	38.96–45.42
Mb	x̄ ± Std. Err	1.0 ± 0.30	0.09 ± 0.02	1.02 ± .2	0.50 ± 0.24	0.16 ± 0.06	1.66 ± .48	13.5 ± 4.5	39.1 ± 14.72
	Range	0.47–1.5	0.06–0.12	0.7–1.42	0.02–0.75	0.05–0.23	0.76–2.4	5.6–21	11.2–61.2

Ammonia- nitrogen (NH_3_-N) concentrations ranged from 0.17 to 0.29 mg L^−l^ in dry and 0.08 to 0.15 mg L^−1^ in wet seasons with the lowest concentrations at C and M_a_ in dry and K_o_ and M_a_ in wet seasons while the highest values in F_b_ and K_b_ in dry and wet seasons respectively (Tables 6 and 7). The mean total nitrogen (TN) concentrations ranged from 5.69 to 12.21 mg L^−1^ in dry and 4.98 to 12.0 mg L^−1^ in wet seasons. The highest concentrations were at K_o_ in dry and at F_b_ in wet season whereas the lowest concentrations were at B in both seasons (Tables 6 and 7).

Soluble reactive phosphorus (SRP) ranged from 0.05 to 0.08 mg L^−1^ and showed similar concentrations for lower values for most of the sampling sites and high values at F_b_ in the dry season, while in the wet season it ranged from 0.05 to 0.12 mg L^−1^ (Tables 6 and 7). Most sites have also similar concentrations in the wet season and only Site F_b_ had highest values. Similarly, the mean TP concentrations ranged from 0.12 to 0.97 mg L^−1^ and 0.23 to 1.02 mg L^−1^ in dry and wet seasons respectively (Tables 6 and 7). Mean TP concentration was highest at M_b_ in both seasons whereas the lowest concentrations were at B and K_o_ in dry and K_a_ in wet seasons.

The concentration of SiO_2_-Si ranged from 39.4 to 91.3 and 35.5 to 42.6 mg L^−1^ with mean values of 55.4 and 38.5 mg L^−1^ in dry and wet seasons, respectively (Tables 6 and 7) whereas the highest and lowest concentrations were noticed during the dry and wet seasons, respectively (Tables 6 and 7). Significant fluctuations in the mean SiO_2_-Si concentrations were observed in both seasons showed that the fluctuations in it over the different seasons and across the different sampling sites were significant in the lake.

### Multivariate analysis

#### Principal component analysis (PCA)

Four components of PCA analysis showed 88.10% of the variance in the data set of the wet season, as the eigenvectors classified the 15 physico-chemical parameters into four groups. PC_1_ (38.93% of the total variance in the data set) has strong positive loadings on TP, NH_3_-N, NO_2_-N, NO_3_-N, TIN, pH and SD (Table 8). The second component (PC_2_) accounted for 24.02% of the total variance measured, demonstrated strong positive loadings for TN, EC, TDS and TA and the third component (PC_3_) demonstrated 16.76% of the total variance and have strong positive loadings on SiO_2_-Si, PO_4_-P, DO and temperature, while, the fourth component (PC_4_) accounts only 8.39% of the total variance in the season (Table 8).

**Table 8:** The Factor loadings values and explained variance of water quality in two seasons (positive and negative strong correlations are marked bold)

Dry season					Wet season				
Parameters	PC1	PC 2	PC3	PC 4	Parameters	PC1	PC2	PC3	PC 4
TP	0.065	**− 0.69**	0.48	0.10	TP	**0.92**	**− **0.18	0.21	0.23
PO4	**0.93**	**− **0.16	0.13	**− **0.03	PO4	0.15	**− **0.18	**0.85**	0.37
NH3	**0.907**	**− **0.04	0.27	**− **0.11	NH3	**0.85**	**− **0.38	0.32	0.05
NO2	**0.966**	0.10	**− **0.07	**− **0.09	NO2	**0.88**	0.09	**− **0.33	**− **0.15
NO3	**0.963**	**− **0.04	**− **0.21	0.10	NO3	**0.90**	**− **0.005	0.03	**− **0.34
TIN	**0.98**	**− **0.01	**− **0.17	0.06	TIN	**0.94**	0.03	**− **0.14	**− **0.235
TN	**− **0.097	**− **0.32	0.18	**0.88**	TN	0.56	**0.68**	**− **0.28	0.20
SiO2	**0.791**	**− **0.52	**− **0.29	**− **0.05	SiO2	0.11	**− **0.10	**− 0.62**	0.49
Temp	0.283	**0.79**	0.43	**− **0.09	Temp	**− **0.11	0.19	**0.63**	**− **0.62
DO	0.349	**− **0.42	**0.76**	**− **0.09	DO	0.08	0.55	**0.64**	0.40
PH	**− **0.373	**0.76**	0.49	0.08	pH	**− 0.85**	0.13	0.01	**− **0.17
EC	**0.963**	0.16	**− **0.02	0.09	EC	**− **0.25	**0.96**	**− **0.07	0.03
TDS	**0.955**	0.17	**− **0.05	0.08	TDS	**− **0.26	**0.95**	**− **0.087	0.03
SD	0.035	**0.66**	**− **0.34	0.32	SD	**0.61**	0.58	**− **0.21	**− **0.16
TA	**0.801**	0.54	0.20	0.11	TA	0.44	**0.63**	0.43	0.03
Eigen value	7.97	3.05	1.66	0.96	Eigen value	5.84	3.60	2.52	1.26
% variance	53.133	20.34	11.09	6.41	% variance	38.93	24.02	16.76	8.39
% Cumulative variance	53.133	73.47	84.56	90.97	% cumulative variance	38.93	62.95	79.71	88.10

**Fig. 2:**
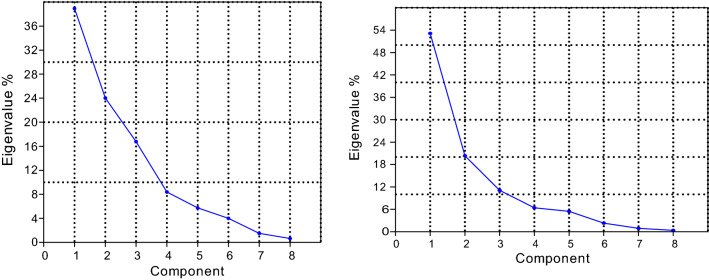
**a** Wet season Scree plot of the eigenvalues. **b** Dry season Scree plot of the eigenvalues

The dry season PCA analysis showed that four principal components (PCs) represented about 90.97% of the total variation in the entire dataset. The first PC accounted for 53.4% of the total variations between sites and comprised of the following parameters: nutrients (NH_3_-N, NO_2_-N, NO_3_-N, TIN, PO_4_-P, SiO_2_-Si), TDS, EC, TA. The second PC accounted for 20.34% of the total variance and had strong positive loading with temperature, pH, TP and SD as the associated parameters. The third PC explained 11.09% of the total variations between sites comprising only DO. Scree plot showed the eigenvalues sorted from large to small as a function of the principal components number after the fourth PC. After the fourth PC (Fig. 2a, b), starting in the downward curve, other components can be omitted.

**Fig. 3:**
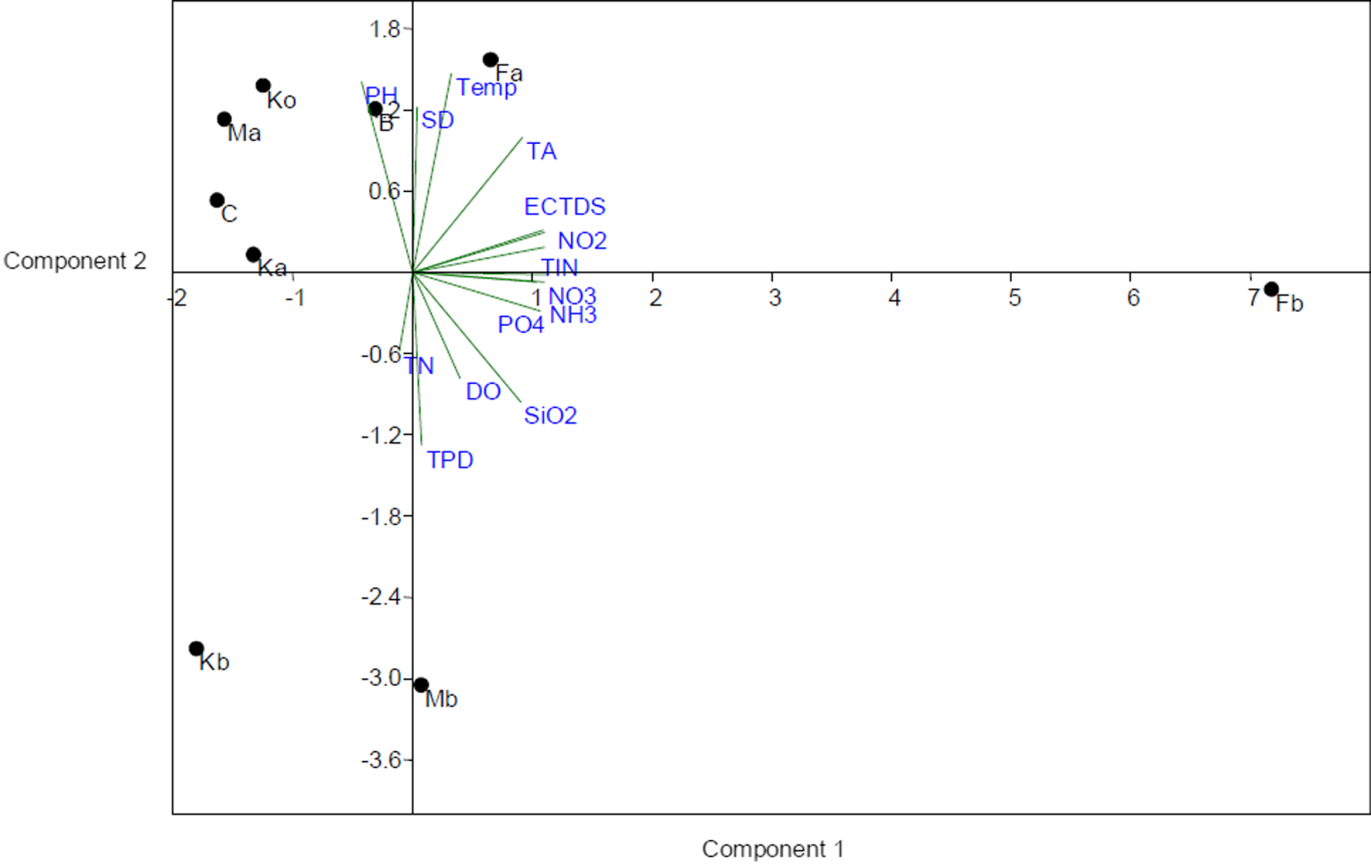
Results of the bi-plot of the correlation between for various water quality parameters with respect to studied sites using PCA in the dry season

The bi-plot of PCs associated with nutrients (NH_3_-N, NO_3_-N, NO_2_-N, SiO_2_-Si and PO_4_-P), EC and TDS characterizing Fb sampling site from axis 1 (Fig. 3) and Fa distinctiveness was attributed to temperature, SD and TA. The parameter influencing the distinction in the B site from axis 2 was mainly pH while Mb site from axis 2 was influenced by DO, TN and TP in the dry season.

**Fig. 4:**
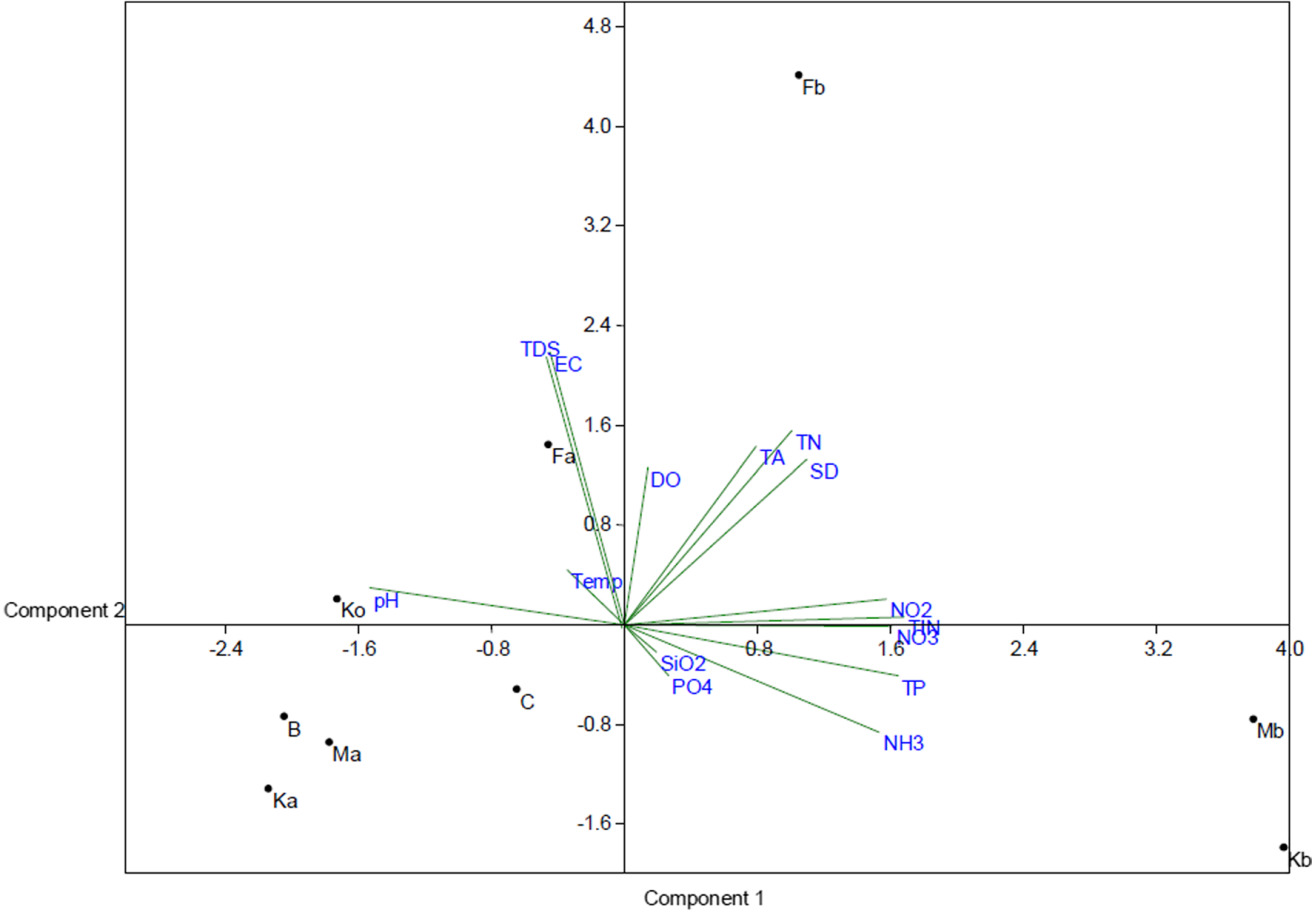
Results of the bi-plot of the correlation between for various water quality parameters with respect to studied sites using PCA in the wet season

The bio-plot of PCs associated with nutrients (NH_3_-N, NO_3_-N, NO_2_-N, SiO_2_-Si, TIN, PO_4_-P and TP), which were the key parameters characterizing the Mb and Kb sampling sites (Fig. 4) and Fa distinctiveness was attributed to temperature, TDS, EC and DO. The parameter influencing the distinction in the K_o_ site was mainly pH while Fb site was influenced by DO, TN, TA and SD in the wet season.

For the two temporal clusters, 90.97% and 88.10% of the variances in dry and wet seasons were explained by the four main factors, respectively.

#### Cluster analysis (CA)

**Fig. 5:**
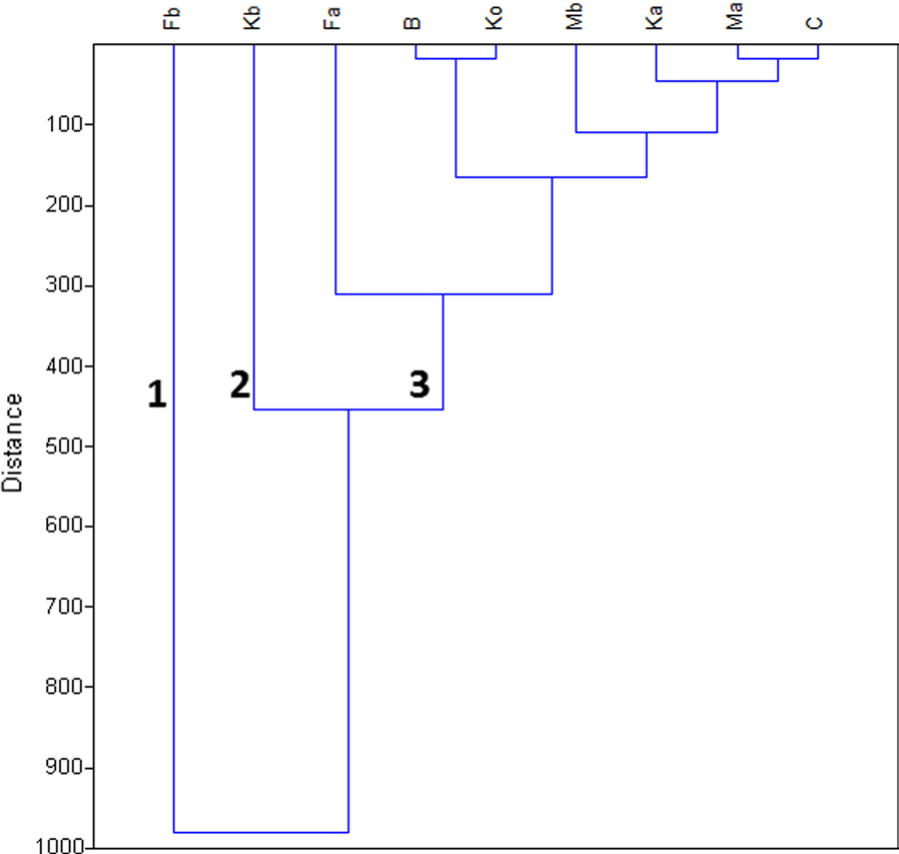
Dendrogram based for agglomerative hierarchical clustering (wards method) based on the PCA scores in dry season

A dendrogram of sampling sites obtained by Ward’s method is shown in Fig. 5. Nine sampling sites were divided into three groups. Cluster 1 corresponded to site Fb, which was located in the western part of the lake. Cluster 2 included site Kb, which were located in the eastern portion of the lake. Cluster 3 contained sites Fa, K_o_ and B the western part of the lake, C which was in the lake central station; site Mb and Ma in northern part of the lake and Ka was in the eastern part of the lake.

**Fig. 6:**
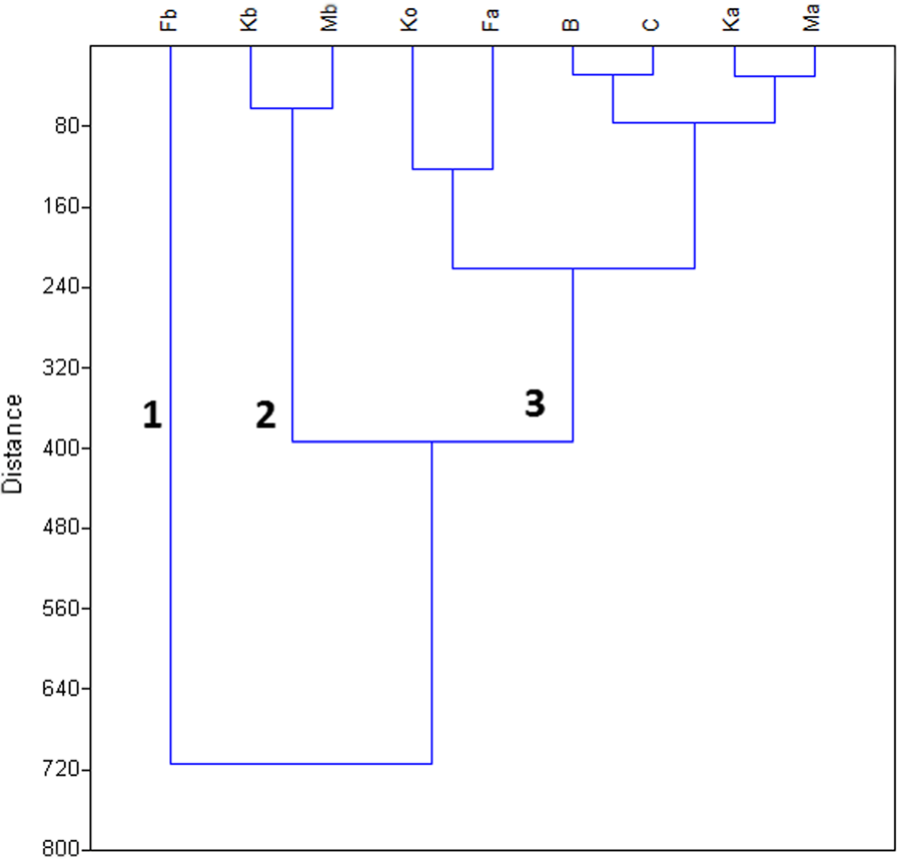
Dendrogram based for agglomerative hierarchical clustering (wards method) based on the PCA scores in the wet season

The wet season classification performed by the use of cluster analysis grouped in all the nine sampling sites of the basin into three statistically significant clusters (Fig. 6).

### Comprehensive evaluation of Lake Ziway water quality analysis

The values of the comprehensive pollution index were 1.8, 1.0, 1.01 and 1.08 for sites Fb, Fa, B and Mb, respectively (Table 9), which demonstrated moderate pollution in dry season while sampling sites of Ka, Ma, C, and Kb have pollution index of 0.71, 0.69, 0.81, 0.79 and 0.84, respectively, which demonstrated slight pollution in the same season. However, in the wet season, the values of the comprehensive pollution index ranged from 0.38 to 0.68 which demonstrated slight pollution of the whole sampling sites.

**Table 9:** Paired samples test for dry and wet seasons

Paired samples test		Paired differences			Sig. (2-tailed)
		Mean	Std. deviation	Std. error mean	
Pair 1	TP in dry—TP in Wet	− 0.20	0.15	0.05	0.00
Pair 2	PO_4_-P in dry—PO4-P in wet	− 0.01	0.02	0.01	0.27
Pair 3	NH_3_-N in Dry—NH3-N wet	0.11	0.05	0.02	0.00
Pair 4	NO_2_-N in Dry—NO2-N in wet	− 0.171	0.502	0.17	0.34
Pair 5	NO_3_-N in Dry—NO3-N in wet	0.48	1.68	0.56	0.42
Pair 6	TIN in dry—TIN in wet	0.41	2.14	0.71	0.58
Pair 7	TN in dry—TN in wet	0.57	2.79	0.93	0.56
Pair 8	SiO_2_-Si in dry—SiO2- Si in wet	16.88	15.68	5.23	0.01
Pair 9	Temp in dry—Temp in wet	1.68	1.22	0.416	0.00
Pair 10	DO in dry—DO in wet	0.58	0.60	0.20	0.02
Pair 11	PH in dry—DO in wet	2.97	0.97	0.32	0.00
Pair 12	EC in dry—EC in wet	109.04	112.82	37.61	0.02
Pair 13	TDS in dry—TDS in wet	73.10	71.67	23.89	0.02
Pair 14	SD in dry—SD in wet	7.73	2.11	0.70	0.00
Pair 15	TA in dry—TA in wet	93.65	85.24	28.41	0.01

### Temporal variation of water quality

Significant temporal variations were observed in physico-chemical parameters and nutrients of Lake Ziway water quality where most of the physicochemical parameters have significantly higher values in the dry season as compared to wet season (P < 0.05) (Table 10).

**Table 10:** Comparison of the physico-chemical parameters and nutrients of Lake Ziway with other tropical lakes (mg L^−1^ for nutrients

Lakes	Temp(^o^c)	DO	P pH	EC	SRP	TP	NO_3_-N	SiO_2_-Si	SD	References
Hawasa	23.5	5–7	8.66	846	0.015	0.034	0.025	37.6	0.85	[30]
Chamo	26.3	5–9	8.84	1910	0.118	0.182	0.033	1.0	0.18	[30]
Hayq	18.2	1–8.4	9	910	0.022	0.058	0.042	3.7	2.7	[46]
Tana	20–27	5.9–7	7.3–8.5	115–148	1.8		0.1–1		0.51–1.82	[58]
Abaya	–	–	8.9	623	0.04	–	–	40	–	[59]
Langano	–	–	9.4	1810	0.09	–	–	48	–	[59]
Bishoftu	–	–	9.2	1830	0.005 to 0.1	–	–	38	–	[59]
Abijata	–	–	10.2	15,800	0.05	–	–	128	–	[59]
Shala	–	–	9.9	19,200	0.76	–	–	112	–	[59]
Chitu	–	–	9.8	28,600	1.7	–	–	320	–	[59]
Ziway	23	5	8.1	404	0.06	0.311	0.21	40.7	0.2	Present study

## Discussion

### Spatial and temporal variations Physico-chemical water quality of Lake Ziway

The spatial and temporal variation of mean water temperature in Lake Ziway was not significant (p > 0.05) during the study period. The mean temperature of the lake water was 23.0 °C in both seasons, which is almost similar to the previously reports in [30] but lower than the value reported by [31]. Lake Ziway has narrow seasonal fluctuations in water temperature due to the lake is shallow tropical lake.

The lowest DO values in dry season at K_o_ was attributed to human impacts like fishing, car and human washing while the low DO level at Kb in the wet season was attributed to its muddy water with agricultural runoff. The highest values of DO at sampling sites B in dry season might be attributed to the presence of macrophytes and phytoplankton with higher biomass and abundance than other sites [31]. The high values of DO at sampling site Fb in the wet season could be probably due to high dilution. The overall mean DO concentration in this study (5.00 mg L^−1^) is much lower than the value reported by [32], (8.72 mg L^−1^). Reference [33] has also reported the DO concentration of 1.4 mg L^−1^ around the floriculture effluent which is smaller than the present study. Concentrations below 4.0 mg L^−1^ adversely affect aquatic life [34]. The value of DO in this study is within the [35] and [36] permissible limits. According to [35] and [36], the standard for DO value for fisheries and aquatic life is between 5.0 to 9.0 mg L^−1^ (Table 10).

The overall mean pH value of the lake water was 8.10 which is in a close agreement with previous data reported by [32] (8.39), [30] (8.65) and [31] (8.44), respectively. However, significant temporal variation was noted during the study as significantly lower value was measured during the rainy season than dry season. The pH value could mainly be controlled by freshwater swamp exudates that regulate the acidity of the water body. A pH range of 6 to 8.5 is normal according to the [18]. In general, the pH of Ziway Lake water is within the acceptable range according to [36] (Table 10).

The overall mean value of EC (404.30 μS cm^−1^) was comparable with previous report of [30, 31, 37] with EC values of 410, 478, 419.14 μS cm^−1^, respectively. Higher conductivity values were measured at the floriculture farming sites than other sampling sites could be attributed to the use of high amount of dissolved agrochemicals from effluents of floriculture industry [33, 38]. For the present study, the EC values of different sampling sites were well below the WHO guideline values prescribed for drinking water purpose (1500 μS cm^−1^) [36]. Accordingly, the value of EC in different water samples could not be water quality problem of the study area. TDS also followed the same trend as that of EC as EC is sensitive to variations in dissolved solids, mostly mineral salts, and there were significantly lower value of EC and TDS during the main rainy season which may be because of dilution.

Similar result of mean SD values with this study (0.21 m) was reported by [39] which was 0.19 m. however, the range values of this study (0.20 to 0.22 m) was smaller than the values which were ranged from 0.20 to 0.35 m and 0.4 to 1.06 m reported by [40, 41], respectively (Table 11). Moreover, [40] also reported that the mean SD value was 0.29 m in Lake Ziway. The declining trend in SD reading is one of the indications which suggest the increasing trend in turbidity of the lake, which can be mainly attributed to catchment degradation and siltation.

**Table 11:** Single pollution index and comprehensive pollution index of nine sampling sites in some selected water quality parameters in dry and wet seasons

Site	Dry season						Wet season					
	P_PO4_	P_NH3_	P_NO2_	P_NO3_	P_DO_	P	P_PO4_	P_NH3_	P_NO2_	P_NO3_	P_DO_	P
Fb	0.83	0.20	1.91	0.53	1.20	1.80	0.60	0.06	0.99	0.53	1.21	0.68
Fa	0.48	0.16	1.06	0.04	1.09	1.0	0.50	0.06	0.93	0.04	1.02	0.51
B	0.55	0.14	0.53	0.02	1.33	1.01	1.10	0.07	0.37	0.02	1.18	0.55
Ka	0.50	0.14	0.39	0.01	0.89	0.71	0.50	0.06	0.52	0.01	0.78	0.38
Ma	0.51	0.11	0.31	0.01	0.88	0.69	0.57	0.05	0.84	0.01	0.84	0.46
Ko	0.51	0.13	0.36	0.01	1.07	0.81	0.50	0.05	0.47	0.01	1.03	0.41
C	0.46	0.11	0.32	0.03	1.04	0.79	0.51	0.06	0.82	0.03	0.97	0.48
Kb	0.52	0.12	0.37	0.02	1.10	0.84	0.59	0.10	1.32	0.02	0.70	0.55
Mb	0.62	0.15	0.45	0.03	1.43	1.08	0.85	0.10	1.13	0.03	1.25	0.67

Reference [40] reported that the mean value of TA in the Lake was 247.5 mg CaCO_3_ L^−1^ which was similar value in this study in dry season (239.3 mg CaCO_3_ L^−1^) where as the value in the wet season (154.6 mg CaCO_3_ L^−1^) was very low. In the lake, TA was solely due to bicarbonates and carbonate alkalinity that could be traced at any station during the entire period of study. According to [7] nutrient status classifications using TA, Lake Ziway can be considered nutrient rich. During all the seasons, fluctuations in TA across the sites were significant. TA has generally decreased in the wet seasons probably due to the dilution effect of the rains and fresh incoming runoffs [42, 43].

### Nutrients analyses

All the nutrient species analyzed in the surface water of the lake showed increased trend. The mean nitrate nitrogen values found in this study (0.21 mg L^−1^) was higher than those values 0.17, 0.003, 0.06 mg L^−1^ reported by [30, 31, 44], respectively. The increasing trend in nitrate concentration in the lake is probably because of nutrient enrichment of the littoral zone of the lake from anthropogenic sources from the catchment area. The mean nitrite nitrogen values found in this study (0.5 mg L^−1^) was also higher than the values reported by previous studies on the lake. For instance, [45] and [31] has reported 0.06 and 0.01 mg L^−1^ nitrite nitrogen respectively. Relatively higher nitrite concentrations were measured near effluent of floriculture industry which could be due to the application of high amount of agrochemicals (Tadele, 2012). Comparatively, higher concentration of nitrite nitrogen value also measured in Lake Ziway than some other Ethiopian lakes for instance, Lake Hayq [46]. The mean concentration of nitrite nitrogen in this study is beyond the concentration limit of the EU guide lines for drinking water (0.1 mg nitrite nitrogen L^−1^) [35]. Consequently, it might cause environmental concern due to its toxicity to aquatic biota as well as because of human health effects.

The mean concentration of NH_3_-N (0.121 mg L^−1^) in this study is closely similar with relatively recent reports; by [39] (0.111 mg L^−1^), and [31] (0.143) but higher than that of earlier reports; for example, by [44] (0.036 mg L^−1^) indicating increasing trend. The mean TN concentration in both dry and wet season in this study is higher than the standard limit value even for eutrophic waters [18] (Table 10).

In addition, the mean SRP concentration (0.06 mg L^−1^) was higher than that of the pervious reported data of [31, 37, 39, 44] which was 0.016, 0.01, 0.059 and 0.029 mg L^−1^, respectively. The measured concentration is also beyond the range of its threshold (0.05 to 0.1mgL^−1^) as a nutrient for natural waters [47, 58]. This is because in recent times Lake Ziway is exposed to anthropogenic activities due to over usage of agrochemicals like fertilizers, pesticides in which organic and inorganic pollutants releases and discharge of water from domestic sources, agricultural runoff, and horticulture including floriculture activities around the lake. Besides, the mean TP value of the lake water (0.311 mg L^−1^) is higher than the previous reported data of [44], and [30], which was 0.069 and 0.219 mg L^−1^, respectively. Higher TP concentration was also measured in this lake in this study as compared to that of other Ethiopian rift valley lakes like Lake Awasa and Chamo [30]. The increasing trend in TP is also probably because of nutrient enrichment of the lake from the highly agricultural activities around the lake watershed [17].

Higher concentration of SiO_2_-Si was found in dry season compared to wet season, this might be because of dilution in the wet season. Similar results were reported by [48]. Significant fluctuations in mean SiO_2_-Si concentrations were observed over the different seasons and across the different sampling sites in the Lake. The range and mean concentration of SiO_2_-Si in this study (39.36 to 91.29 and 35.53 to 42.62 mg L^−1^ with mean values of 55.4 and 38.5 mg L^−1^ in dry and wet seasons, respectively) is higher than that of the reports by previous studies. [37, 59] reported that SiO_2_-Si concentrations of Lake Ziway ranged 13.4 to 31 and 14.7 to 37.5 mg L^−1^ with mean values of 19.0 and 22.9 mg L^−1^ in dry and wet seasons, respectively.

The overall mean concentration of SiO_2_-Si (40.68 mg L^−1^) in this study was higher than the previous reported values in the same lake and other Ethiopian rift valley Lakes, Awasa and Chamo by [30, 58, 59] which was 23.8, 37.6 and 1.00 mg L^−1^ in Lake Ziway, Awasa and Chamo, respectively. In view of the high silica concentrations (> 10 mg SiO_2_ L^−1^) commonly encountered in African lakes [49], the lake might encounter some ecological changes especially towards higher Diatom productivity (Table 10).

Generally, a pattern of low mean concentrations of NH_3_-N, NO_3_-N, TN, TIN, SiO_2_-Si in dry season have higher mean concentrations in wet season. This strongly indicated point source pollution for this parameter, which might be associated with industrial effluents, human interference, municipal discharge and animal waste [14]. During dry season both decreased precipitation and increased agricultural withdraws for irrigation contributed to lower flows of those nutrients, however, TP, PO_4_-P and NO_2_-N were observed in a higher concentration during wet season. Similarly [50, 58], noted that nutrients that have a high concentration during dry season than wet season tend to come from point sources whose supply is constant, whereas the inverse pattern can be attributed to non-point sources that are mobilized by high run-off during wet periods.

### Multivariate analysis

#### Principal component analysis (PCA)

As indicated in the PCA analysis, PC_1_ has strong positive loadings on NH_3_-N, NO_2_-N, NO_3_-N, PO_4_-P, TP, SiO_2_-Si, TIN, EC, TDS, TA and SD associated sampling sites Mb and Kb during wet season. The presence of nutrients in PC_1_ demonstrated the intense of agricultural activities in the environment of the lake ecosystem and this resulted in pollution with nutrients coming from fertilizers and pesticides [14].

One of the main sources of TP in runoff is soils with high phosphorus levels. In other words, the nutrient parameters, pH and SD account for similar patterns seen in lake water samples. This group of nutrient parameters also reflected the degree of eutrophication of the lake, suggesting that the anthropogenic pollution mainly from the discharge of domestic and agricultural wastes, industrial sewage and agricultural runoff [14]. Moreover, it might be due to farmers use ammonium fertilizers and phosphate pesticides, and the lake receive ammonium via surface runoff and irrigation waters [17]. Nitrate nitrogen source is due to numerous sources, such as, geologic deposits, natural organic matter decomposition and agricultural runoff [51]. The second component (PC_2_) demonstrated strong positive loadings for TN, EC, TDS and TA. The third components (PC_3_) demonstrated strong positive loadings for SiO_2_-Si, PO_4_-P, DO and temperature. This factor indicates that PO_4_-P source is from domestic and agricultural wastes, detergents from industries whereas SiO_2_-Si is from bed rock materials and compounds containing silica from floriculture industry [33], while, the fourth component (PC_4_) had no strong loadings in any measured parameters.

In the dry season the PCA performed on the correlation matrix of means of the analyzed water quality parameters by sites showed that four principal components (PCs) represented about 90.97% of the total variation in the entire dataset. The first PC accounted nutrients (NH_3_-N, NO_2_-N, NO_3_-N, TIN, PO_4_-P, SiO_2_-Si), TDS, EC, TA associated with Fa and Fb sampling sites. The high values in these sampling sites were attributed to the point pollution sources from floriculture industry in dry season.

The second PC had strong positive loading with temperature, pH, TP and SD as the associated parameters. TP demonstrating that intense agricultural activity had occurred at the sampling site Fa and B, causing pollution due to fertilizers and pesticides [14, 42] interpreted as nutrient pollution from anthropogenic sources, such as eutrophication from domestic wastewater, industrial effluents and agricultural activities. The third PC explained the total variations between sites comprising only DO in sampling site Mb. The inverse relationship between temperature and DO is a natural process because it can hold less dissolved oxygen [42]. The fourth PC explained site variations with TN only. [52] classified the factor loadings as “strong,” “moderate,” and “weak,” corresponding to absolute loading values of > 0.75, 0.75 to 0.50, and 0.50 to 0.30, respectively.

In the present study, a scree plot also showed the eigen values sorted from large to small as a function of the principal components number. After the fourth PC (Fig. 2a, b), starting in the downward curve, other components can be omitted. The scree plot was used to identify the number of PCs to be retained in order to comprehend the underlying data structure [53]. Thus, a new set of data is obtained. This may explain the variation of data set with fewer variables. Scree plots in PCA/FA to visually assess which components or factors explain most of the variability in the data.

The bi-plot of PCs associated with nutrients (NH_3_-N, NO_3_-N, NO_2_-N, SiO_2_-Si and PO_4_-P), EC and TDS which were the key parameters characterizing the Fb sampling site (Fig. 3), which were due to the floriculture effluents [31, 33] and Fa distinctiveness was attributed to temperature, SD and TA. The parameter influencing the distinction in sampling site B was mainly pH while Mb site was influenced by DO, TN and TP in the dry season [31].

The bio-plot of PCs associated with nutrients (NH_3_-N, NO_3_-N, NO_2_-N, SiO_2_-Si, TIN, PO_4_-P and TP), which were the key parameters characterizing the Mb and Kb sampling sites (Fig. 4), can suggest an influence of agricultural activities in the catchment of the two rivers feeding the lake (Meki and Ketar Rivers) and Fa distinctiveness was attributed to temperature, TDS, EC and DO. The parameter influencing the distinction in the K_o_ site was mainly pH while Fb site was influenced by DO, TN, TA and SD in the wet season.

The results from temporal PCA/FA suggested that agrochemicals pollution were potential pollution sources for both temporal clusters but that the influence of each was different. The results of the present study showed the existence of the contamination of Lake Ziway in both inorganic and organic agrochemicals mainly in the lake catchment in particular to Fb, Fa, Mb and Kb sampling sites. The major pollutant sources to the lake might be mainly from agricultural activities, human interference for different purposes, domestic wastes, industrial effluents and urban origin [14].

#### Cluster analysis (CA)

The three groups obtained by cluster analysis vary according to natural backgrounds features, land use and land cover, industrial structure and anthropogenic sources of pollution [14]. The cluster analysis revealed different properties at each site with respect to physical and chemical variables.

Sites mainly located at middle reach of the lake (Station C, M_a_, K_a_, K_o_ and F_a_) were grouped under Cluster III, which were basically at the center of the lake and shore water. In addition, Station Ma and Ka located upstream of the lake, showed the similar water environment quality characteristics with these stations. Urbanization and industrialization level is relatively low at these sites. Direct discharged domestic wastewater contaminated the water; the cluster III correspond to relatively less polluted (LP), because the inclusion of the sampling location suggests the anthropogenic sources of pollution is less in the study period.

Mb and Kb sampling sites were grouped under cluster II; the two stations are the tributaries of the lake; one is Meki River that drains part of the western high land and the second is the Ketar River which can drains the Arsi Mountains to the eastern part of the lake. These two rivers transported many agrochemicals from western high land and Arsi Mountains ([14, 17]. Therefore, these sampling stations received pollutants mostly from agricultural runoff, domestic waste and industrial effluent from the local people and Meki and Abura towns ([14, 17]. Cluster II corresponds to moderately pollution.

Sampling site Fb is grouped under Cluster I; this cluster site is the effluents of the floriculture industries which is directly enter to the lake and polluted the lake water. Cluster I correspond to relatively highly polluted (HP) site, because the inclusion of floriculture industry, due to the untreated sewage of floriculture effluent at this site [33]. Accordingly, spatial variations of water quality in Lake Ziway showed that water quality was better in center and some portions of the shore water than in western and eastern areas in the lake. At the same time these results showed that for a rapid assessment of water quality, only one site in each cluster presents a useful spatial assessment of the water quality for the entire network in different seasons. This implies that, the results indicate the CA technique is useful in offering reliable classification of surface water in the whole region and make it possible to design a future spatial sampling strategy in an optimal method, which can reduce the number of sampling sites and associated costs. Similar reports have been dispatched by different authors [7, 25].

This implies that, for a rapid assessment of water quality, only one site in each cluster presents a useful spatial assessment of the water quality for the entire network in different seasons. In this study we found the PCA and CA analysis techniques are useful in apportionment of pollution sources based on parameter association. Similar findings has been reported in the study of [50, 54–56].

Other water quality studies that applied PCA and CA analysis found the techniques helpful in the interpretation of large datasets. [54] used PCA and CA in the analysis of water quality in Manchar Lake in Pakistan and found the techniques useful in apportionment of pollution sources based on parameter association. Their findings agree with the present study particularly in the association of nutrients with catchment runoff in the wet season and point sources during the dry season. Reference [55] used PCA and CA in 33 sampling sites for 13 physico-chemical and biological water quality parameters which helped them in identifying the underlying processes responsible for the heterogeneity in different parts of Lake Neusiedler in Hungary. Their study also showed that the river input region was significantly different. Moreover, [56], 16 water quality parameters; in their finding also agreed with the present study that most parameters increase their concentrations in the dry season due to evaporative effects, whereas lower values are observed in the wet season as the lake water is diluted by rain water. Furthermore, [50] also applied PCA and CA to identify the factors influencing the water quality in different seasons in Hyderabad lakes in India. The study revealed that water pollution was more significant during the dry season as compared to the rainy season because of precipitation and tidal influence which cause dilution. [57], applied PCA and CA in order to provide an insight on water quality in Lake Naivasha, Kenya showed the usefulness of such multivariate analysis in establishing the characteristics of different regions in aquatic ecosystems based on numerous water quality parameters.

### Comprehensive evaluation of Lake Ziway water quality analysis

According to the comprehensive pollution index values sites Fb, Fa, B and Mb showed moderate pollution in dry season. The low water qualities parameters in these sites might be the influences of floriculture industry and domestic wastes from Ziway and Meki Towns. However, the wet season, the values of the comprehensive pollution index ranged from (0.38 to 0.68) demonstrated slight pollution of the whole sampling sites. The water quality of the lake was determined to have been influenced by different major source of pollution such as agricultural activities, domestic wastes, fishing industries, swimming and car washing. Similarly [24], applied comprehensive pollution index model to explain the pollution status of Lake Baiyangdian, china. Their result revealed that Lake Baiyangdian has a pollution status ranging from less polluted to sever polluted.

## Conclusion

Lake Ziway has shown some undesirable changes in terms of hydrology and lake water quality due to uncontrolled agrochemicals use in the lake watershed. The concentrations of most physicochemical parameters and nutrients showed high values in dry season and then decreased in wet season. All the nutrient species analyzed in the surface water of the lake showed increased trend and these variables might be primarily due to different environmental factors associated with intensive anthropogenic activities in the lake catchment specially, in western and eastern zones of the lake. The increasing trend in nutrient levels and in some water quality parameters in this study may lead to long term ecological changes in the lake ecosystem unless possible measures should be taken. In order to stop further deterioration of the lake water quality and to eventually restore the beneficial uses of the lake, management of phosphorus and nitrogen load and the appropriate fertilizer use should be a priority in the lake watershed. Most of all free access policy (no ownership scenario) to water bodies may have to change for Lake Ziway by giving concession rights to users with the appropriate environmental regulatory protocols. Understanding this variation may help in developing mitigation and restoration strategies for the lake and aquatic ecosystems in Ethiopia.

## Data Availability

The datasets used and/or analyzed during the current study are available from the corresponding author.
